# PairMotifChIP: A Fast Algorithm for Discovery of Patterns Conserved in Large ChIP-seq Data Sets

**DOI:** 10.1155/2016/4986707

**Published:** 2016-10-24

**Authors:** Qiang Yu, Hongwei Huo, Dazheng Feng

**Affiliations:** ^1^School of Computer Science and Technology, Xidian University, Xi'an 710071, China; ^2^School of Electronic Engineering, Xidian University, Xi'an 710071, China

## Abstract

Identifying conserved patterns in DNA sequences, namely, motif discovery, is an important and challenging computational task. With hundreds or more sequences contained, the high-throughput sequencing data set is helpful to improve the identification accuracy of motif discovery but requires an even higher computing performance. To efficiently identify motifs in large DNA data sets, a new algorithm called PairMotifChIP is proposed by extracting and combining pairs of *l*-mers in the input with relatively small Hamming distance. In particular, a method for rapidly extracting pairs of *l*-mers is designed, which can be used not only for PairMotifChIP, but also for other DNA data mining tasks with the same demand. Experimental results on the simulated data show that the proposed algorithm can find motifs successfully and runs faster than the state-of-the-art motif discovery algorithms. Furthermore, the validity of the proposed algorithm has been verified on real data.

## 1. Introduction

A DNA motif is a conserved pattern occurring in the regulatory region of DNA sequences with small mutations [1]. All occurrences of the motif in the sequences are called motif instances or motif sites, which are usually the sequence fragments with specific biological functions such as transcription factor binding sites (TFBSs) [2]. TFBSs are important regulatory elements that control transcription initiation and transcription efficiency of the associated genes. Identifying motifs in a given set of DNA sequences is the basis for analysis of gene expression regulation [3] and the precursor to identifying disease-associated regulatory variations [4].

Though very important, motif discovery is a challenging computational task. Given a set of DNA sequences, (i) the motif and its instances are unknown; (ii) each of the input DNA sequences is long with hundreds of bases, while the motif is short, generally 5 to 25 bases [5]; (iii) a portion of the input sequences may not contain motif instances; (iv) the input sequences typically contain the disturbance of random overrepresented substrings. In 2003, Evans et al. proved that motif discovery is NP-complete [6]. In addition, with the development of biological experimental techniques, the data used for motif discovery have been changed from traditional promoter sequence data sets to high-throughput sequencing data sets [7]. A traditional data set typically contains only a few to dozens of sequences. A high-throughput sequencing data set is a set of peak regions containing TFBSs obtained through ChIP-seq experiments [8], read mapping [9], and peak calling [10]. It contains hundreds or more sequences, thus forming a large DNA sequence data set, and further increases the difficulty of rapid and accurate identification of motifs.

Currently, there are a lot of motif discovery algorithms to deal with small-scale data sets, such as Weeder [11], PairMotif [12], PairMotif+ [13], MEME [14], PMS8 [15], and qPMS9 [16]; for more algorithms, refer to [7, 17]. Because of high time or space complexity, these algorithms cannot be used for motif discovery in high-throughput sequencing data sets directly.

This paper mainly focuses on motif discovery algorithms for high-throughput sequencing data sets. According to motif representation, the algorithms can be divided into two categories. The algorithms in the first category represent motifs as words. Some of these algorithms, such as F-motif [18] and weeder2 [19], use pattern-driven ideas. They exhaustively verify all possible strings of the motif length over the DNA alphabet and then output the strings that satisfy specified motif property. When verifying motifs, F-motif and weeder2 use the suffix tree and De Bruijn graph techniques, respectively. Some other algorithms, such as RSAT [20], CisFinder [21], and MCES [22], adopt word counting ideas; namely, they mine the substrings in input sequences with high occurrence frequency and then combine them into motifs. Besides a test set, these algorithms often require a control set to eliminate the disturbance of random overrepresented substrings.

The second category covers the discovery algorithms representing motifs as position weight matrixes (PWMs). A set of aligned sites of the same length in the input sequences can form a PWM. These algorithms often select some initial PWMs with certain means and then update each PWM iteratively until it reaches the maximum score. MEME-ChIP [23] is a well-known motif discovery algorithm for ChIP-seq data sets, which updates initial PWMs using the expectation maximization method. STEME [24], another discovery algorithm based on expectation maximization, uses suffix trees to improve the time performance of motif discovery when implementing expectation maximization. Currently, there is no discovery algorithm completely superior to others, and thus, in order to tackle false positives produced by individual discovery algorithms, ensemble algorithms [25] integrate multiple existing discovery algorithms to improve the quality of identified motifs.

In order to efficiently identify motifs in large DNA data sets, we propose a new algorithm, which identifies motifs by extracting and combining pairs of *l*-mers in the input with relatively small Hamming distance. Comparisons with the state-of-the-art motif discovery algorithms show that the proposed algorithm can find motifs successfully with the shortest running time. Also, the validity of the proposed algorithm has been verified on real data.

## 2. Materials and Methods

### 2.1. Algorithm Overview

The notations frequently used in this paper are summarized in the Notations. When we say a pair of *l*-mers, we are referring to two *l*-mers that come from two distinct sequences.

Almost all de novo motif discovery algorithms make identification based on the fact that the motif instances of the same motif are similar to each other. In other words, the motif information contained in the input sequences is presented by the similarity among motif instances. In addition to the degree of similarity among motif instances, the motif information also depends on the number of pairs of motif instances contained in the input sequences, denoted by *N*_mip_. It is calculated by

(1) $$\begin{array}{c} N_{mip}=\frac{qt\left(qt-1\right)}{2}. \end{array}$$

In our previous work, PairMotif [12] and PairMotif+ [13], we mainly process promoter sequences, which correspond to a small *t*. The basic idea is to extract some pairs of *l*-mers in the input, making them contain at least one pair of motif instances, and then refine each pair of *l*-mers to get motifs. Because of the small value of *N*_mip_, limited motif information can be obtained while retaining a large amount of disturbance information. Thus, in order to ensure good identification accuracy, exhaustive methods based on pattern-driven ideas are used for refinement, which has a poor time performance.

In the current work, we propose a new algorithm called PairMotifChIP, which is used for processing large DNA data sets. Our basic idea is still to extract pairs of *l*-mers in the input. Since the value of *N*_mip_ under large data sets is significantly greater than that under traditional promoter data sets, the advantages are as follows: (i) the extracted pairs of *l*-mers contain sufficient pairs of motif instances and (ii) it can be easier to filter out most of the random overrepresented pairs of *l*-mers; namely, we can distinguish most of the pairs of motif instances and random overrepresented pairs of *l*-mers by probabilistic analysis (see Section 3.1). Therefore, after extracting pairs of *l*-mers, we perform filtration to filter out most of the random overrepresented pairs of *l*-mers and then combine the remaining *l*-mers using clustering methods to obtain motifs while eliminating other random overrepresented *l*-mers.

The overall algorithm of PairMotifChIP is shown in Algorithm 1, containing three steps: extracting pairs of *l*-mers (lines (2)–(4)), filtering pairs of *l*-mers (lines (5)–(9)), and combining *l*-mers (lines (10)–(13)). Next, the technical details of the three steps are described in detail.

### 2.2. Extracting Pairs of *l*-mers

In this step, we need to determine the value of a threshold *k* so that we extract all pairs of *l*-mers *x* and *x*′ in the input satisfying *d*_*H*_(*x*, *x*′) ≤ *k* and design an efficient algorithm to extract pairs of *l*-mers.

Let *p*_*i*_ denote the probability that the Hamming distance between two random *l*-mers is no more than* i*.

(2) $$\begin{array}{c} p_{i}=\overset{i}{\underset{k=0}{\sum }}\left(\begin{array}{c} l\\ k \end{array}\right)\times \frac{3^{k}}{4^{l}}. \end{array}$$

Let *E*_*i*_ denote the expectation of the number of pairs of *l*-mers *x* and *x*′ in two* n*-length DNA sequences satisfying *d*_*H*_(*x*, *x*′) ≤ *i*.

(3) $$\begin{array}{c} E_{i}={\left(\;n-l+1\;\right)}^{2}\times p_{i}. \end{array}$$

The threshold *k* is determined by (4), aiming at eliminating random overrepresented substrings in the pairs of *l*-mers extracted from two *n*-length sequences.

(4) $$\begin{array}{c} k=\max\left\{\;i:0\leq i\leq l,\;\;E_{i}<1\;\right\} \end{array}$$

In order to handle large data sets efficiently, a good time performance of extracting pairs of *l*-mers is necessary. For extracting pairs of *l*-mers *x* and *x*′ from two* n*-length sequences *s* and *s*′ satisfying *d*_*H*_(*x*, *x*′) ≤ *k*, the simplest way is to traverse each pair of *l*-mers *x* and *x*′ and calculate the Hamming distance *d*_*H*_(*x*, *x*′) separately. The time complexity of this method is *O*(*n* ^2^ *l*). Yu et al. [26] proposed a method of *O*(*n* ^2^) time by filling an *n* × *n* matrix *M*. The element in row *i* (1 ≤ *i* ≤ *n*) and column *j* (1 ≤ *j* ≤ *n*) of *M*, denoted by *M*[*i*, *j*], stores min(*i*, *j*) − *d*_*H*_(*s*[*i* − min⁡(*i*, *j*) + 1 ⋯ *i*], *s*′[*j* − min⁡(*i*, *j*) + 1 ⋯ *j*]), where min(*i*, *j*) is the smaller one of two integers. After calculating *M*[*i*, *j*], the Hamming distance between the two *l*-mers *s*[*i* − *l* + 1 ⋯ *i*] and *s*′[*j* − *l* + 1 ⋯ *j*] can be obtained; namely, *d*_*H*_(*s*[*i* − *l* + 1 ⋯ *i*], *s*′[*j* − *l* + 1 ⋯ *j*]) = *l* − (*M*[*i*, *j*] − *M*[*i* − *l*, *j* − *l*]).

In this paper, we design a more efficient method. We only care about the pairs of *l*-mers with Hamming distance no more than *k*. That is, we do not need to calculate Hamming distance for all pairs of *l*-mers. If the Hamming distance between *s*[*i* − *l* + 1 ⋯ *i*] and *s*′[*j* − *l* + 1 ⋯ *j*] is greater than *k*, it can be concluded that, for 0 ≤ *h* < *d*_*H*_(*s*[*i* − *l* + 1 ⋯ *i*], *s*′[*j* − *l* + 1 ⋯ *j*]) − *k*, the possibly smallest Hamming distance between *s*[*i* − *l* + 1 + *h* ⋯ *i*] and *s*′[*j* − *l* + 1 + *h* ⋯ *j*] is still greater than *k*. Thus, after calculating the Hamming distance between *s*[*i* − *l* + 1 ⋯ *i*] and *s*′[*j* − *l* + 1 ⋯ *j*], we can go directly to verify the Hamming distance between *s*[*i* − *l* + 1 + *H* ⋯ *i*] and *s*′[*j* − *l* + 1 + *H* ⋯ *j*], where *H* is the skipped size calculated by

(5) H=max⁡1,dHsi−l+1⋯i,s′j−l+1⋯j−k.

**Figure 1:**
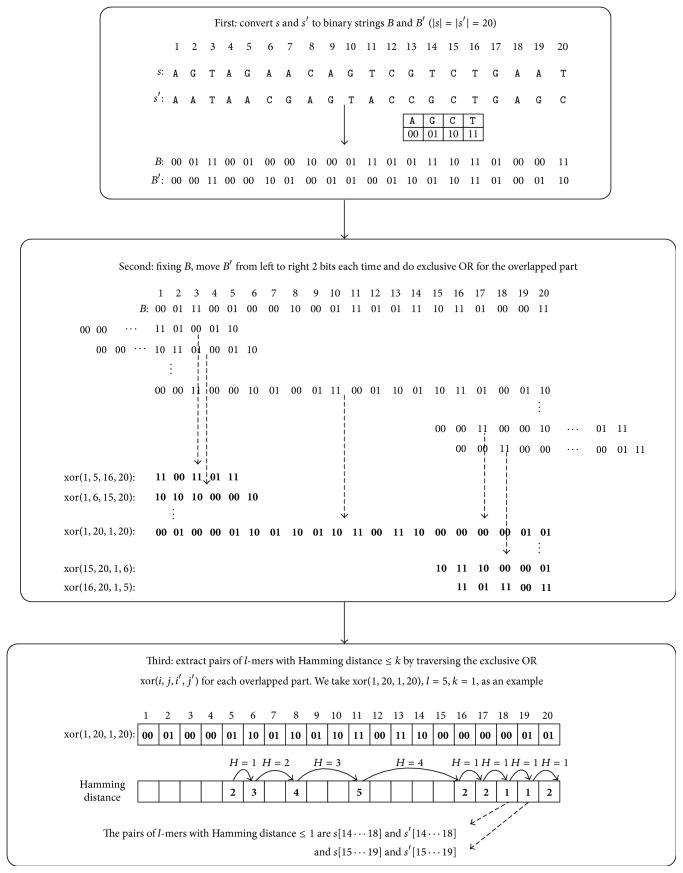
An example for extracting pairs of *l*-mers.

Based on this, we describe our method as follows.  Figure 1 shows an example in which |*s*| = |*s*′| = 20,  *l* = 5,  *k* = 1. First, convert the two DNA sequences *s* and *s*′ into binary strings *B* and *B*′. Then, fixing *B*, move *B*′ from left to right gradually 2 bits each time and simultaneously do the exclusive OR for the overlapped substrings of *B* and *B*′ with length equal to or greater than 2*l*; xor(*i*, *j*, *i*′, *j*′) denotes the exclusive OR of the overlapped substrings of *B* and *B*′ corresponding to *s*[*i* ⋯ *j*] and *s*′[*i*′ ⋯ *j*′], where *j* − *i* = *j*′ − *i*′. Finally, extract pairs of *l*-mers with Hamming distance no more than *k* by traversing the exclusive OR xor(*i*, *j*, *i*′, *j*′) for each overlapped part of *B* and *B*′. Specifically, for each *r* (*l* ≤ *r* ≤ *j*), we look up a table [12] to obtain the Hamming distance between two *l*-mers *s*[*i* + *r* − *l* ⋯ *i* + *r* − 1] and *s*′[*i*′ + *r* − *l* ⋯ *i*′ + *r* − 1], which is equal to the number of 2 bits that is not 00 in xor(*i* + *r* − *l*, *i* + *r* − 1, *i*′ + *r* − *l*, *i*′ + *r* − 1). During the traversal, we use (5) to avoid the calculation for some pairs of *l*-mers with Hamming distance greater than *k*.

### 2.3. Filtering Pairs of *l*-mers

The purpose of this step is to filter those random overrepresented pairs of *l*-mers extracted in the previous step. According to the property of conservation, if an *l*-mer *x* is a motif instance, there may be some other motif instances with Hamming distance no more than *k* from* x*, and thus there may be relatively more *l*-mers in the whole data set with Hamming distance no more than *k* from *x*. If an *l*-mer *x* is not a motif instance, even if it appears in a random overrepresented pair of *l*-mers, there are not many *l*-mers in the whole data set with Hamming distance no more than *k* from *x*.

For an arbitrary *l*-mer *x*, let occ_*r*_(*x*) denote the number of *l*-mers in the input sequences with Hamming distance no more than *k* from *x* in random case.

(6) $$\begin{array}{c} occ_{r}\left(\;x\;\right)=t\times \left(\;n-l+1\;\right)\times p_{k}. \end{array}$$

For an arbitrary motif instance *x*, let occ_*m*_(*x*) denote the number of motif instances in the input sequences with Hamming distance no more than *k* from *x* in random case.

We perform filtration according to (7). Let occ(*x*) denote the number of *l*-mers in the input sequences with Hamming distance no more than *k* from an *l*-mer *x*. In the process of extracting pairs of *l*-mers, we can easily record occ(*x*) for each *l*-mer *x* in the input sequences. For an extracted *l*-mer *x*, we filter it out if it does not satisfy

(7) $$\begin{array}{c} occ\left(\;x\;\right)\geq occ_{r}\left(\;x\;\right)+occ_{m}\left(\;x\;\right). \end{array}$$

In the following, we focus on how to calculate occ_*m*_(*x*). We calculate it by combining the methods [13, 22] for evaluating the following probabilities. One is the probability that the Hamming distance between a motif *m* and a random motif instance *m*′ is *i* (0 ≤ *i* ≤ *d*), denoted by Pr(*d*_*H*_(*m*, *m*′) = *i*). The other is the probability that the Hamming distance between two random motif instances *m*_1_ and *m*_2_ is no more than *k*, denoted by *p*_*k*_′.

Let *d* denote the maximum number of positions where a motif differs from its instance. Given the motif length* l*, we determine the value of *d* in terms of the challenging problem instance of planted motif search [27], which assumes that motif instances are implanted into sequences. When generating a random motif instance *m*′ from a motif *m*, we select *d* out of *l* positions randomly and then make the character at each of the *d* positions change with a probability of *g*, which is the conservation parameter reflecting the conservation degree of a motif. Based on this, Pr(*d*_*H*_(*m*, *m*′) = *i*) is evaluated as follows:

(8) $$\begin{array}{c} Pr\left(\;d_{H}\left(\;m,m^{\prime }\;\right)=i\;\right)=\left(\begin{array}{c} d\\ i \end{array}\right)\times g^{i}\times {\left(\;1-g\;\right)}^{d-i}. \end{array}$$

For any two instances *m*_1_ and *m*_2_ of a motif *m*, the possible values of 〈*d*_*H*_(*m*, *m*_1_), *d*_*H*_(*m*, *m*_2_)〉 are {〈*a*, *b*〉 :  0 ≤ *a* ≤ *d*,  0 ≤ *b* ≤ *d*}. Let Pr(〈*a*, *b*〉) denote Pr(〈*d*_*H*_(*m*, *m*_1_), *d*_*H*_(*m*, *m*_2_)〉 = 〈*a*, *b*〉). We have

(9) Pra,b=PrdHm,m1=a×PrdHm,m2=b.

By the theorem of total probability, *p*_*k*_′ is evaluated using (10) where the value of *P*(*d*_*H*_(*m*_1_, *m*_2_) ≤ *k*∣〈*a*, *b*〉) is calculated in terms of the actual situation [13].

(10) pk′=∑0≤a,b≤dPra,b×PrdHm1,m2≤k ∣ a,b.

Finally, we calculate occ_*m*_(*x*) as follows:

(11) $$\begin{array}{c} occ_{m}\left(\;x\;\right)=t\times q\times p_{k}^{\prime }. \end{array}$$

### 2.4. Combining *l*-mers

After performing filtration, we combine the remaining *l*-mers by using the clustering method. On the one hand, we further eliminate random overrepresented *l*-mers, as the filtration carried out in the previous step cannot guarantee that all the random overrepresented *l*-mers are filtered out. On the other hand, the input data may contain more than one motif, and thus the clustering method is also used to distinguish the instances of different motifs and gather the instances of the same motif together.

The combining method is described in detail as follows:

(i) Merge the overlapped *l*-mers into one substring. For example, for the three *l*-mers *s*[*i* ⋯ *i* + *l* − 1], *s*[*i* + 2 ⋯ *i* + *l* + 1], and *s*[*i* + 5 ⋯ *i* + *l* + 4] in the sequence *s*, they are overlapped, and we merge them into a new substring *s*[*i* ⋯ *i* + *l* + 4].
(ii) Cluster the substrings. At first, we build an undirected graph *G* by taking each substring as a vertex. For any two vertices *v*_1_ and *v*_2_, assume the corresponding substrings are str_1_ and str_2_, respectively. If there exist an *l*-mer *x*_1_ in str_1_ and an *l*-mer *x*_2_ in str_2_ such that *d*_*H*_(*x*_1_, *x*_2_) ≤ *k*, then we set the weight of the edge connected by *v*_1_ and *v*_2_ to 1, otherwise 0. Secondly, we cluster the vertices in the graph *G* using the MCL clustering algorithm [28]. Finally, we merge the obtained clusters using the MCL clustering algorithm again to further eliminate redundancy.
(iii) For each obtained cluster, align the substrings in it and then fetch the fragment with high information content as an identified motif.

In the combining method, we control the number of elements to be clustered in order to ensure a good time performance. First, merging overlapped *l*-mers into one substring can help to reduce the elements to be clustered. Second, if the number of substrings is still large, we divide them into multiple groups with each group containing at most 1000 substrings. Then, we cluster substrings in each group separately and finally merge the obtained clusters.

## 3. Results and Discussion

### 3.1. Probabilistic Analysis of Extracting Pairs of *l*-mers

We use probabilistic analysis to demonstrate the feasibility of motif discovery by extracting pairs of *l*-mers with relatively small Hamming distance for processing large DNA data sets. That is, given *t* DNA sequences, we verify whether the extracted pairs of *l*-mers with Hamming distance no more than *k* contain sufficient pairs of motif instances and whether the pairs of motif instances and the random overrepresented pairs of *l*-mers can be distinguished.

Table 1 shows a set of data for probabilistic analysis. In generating these data, we set the number of sequences *t*, the sequence length *n*, the probability *q* that each input sequence contains a motif instance, and the motif conservation parameter *g* to 1000, 200, 0.8, and 0.8, respectively. For a fixed *l*, *k* is obtained by (4), which is the maximum making *E*_*k*_ smaller than 1. Let *N*_1_ and *N*_2_ denote the number of pairs of *l*-mers and that of pairs of motif instances with Hamming distance no more than *k* contained in *t* *n*-length sequences at random, respectively. The notations occ_*r*_(*x*) and occ_*m*_(*x*) are explained in the Notations.

(12) N1=t2×n−l+12×pkN2=qt2×pk′.

From the values of *N*_1_ and *N*_2_, it can be found that the extracted pairs of *l*-mers with Hamming distance no more than *k* contain sufficient pairs of motif instances and meanwhile many random overrepresented pairs of *l*-mers. For a given *l*-mer *x*, the *l*-mer in the input with Hamming distance no more than *k* from *x* is called a* k*-neighbor of *x*. From the values of occ_*r*_(*x*) and occ_*m*_(*x*), the number of *k*-neighbors of a motif instance is significantly larger than that of random overrepresented *l*-mers. Thus, we can distinguish the pairs of motif instances and the random overrepresented pairs of *l*-mers, so as to filter out most random overrepresented pairs of *l*-mers extracted in step 1. It should be noted that, for Table 1, we set the motif conservation parameter to 0.8, which is in low conservation case; the value of occ_*m*_(*x*) increases with the increase of the conservation degree, which makes it easier to distinguish the pairs of motif instances and the random overrepresented pairs of *l*-mers. In implementing PairMotifChIP, we fix the value of *l* to 15 because it corresponds to a large value of occ_*m*_(*x*).

### 3.2. Data

We use both simulated and real data to make experiments. The simulated data are helpful for the comparison of different motif discovery algorithms, since the motifs and their locations are known exactly. The real data are mainly used to test whether the proposed algorithm can find motifs under the realistic biological situation.

We generate simulated DNA data according to [29]: generate *t* *n*-length sequences and an* l*-length motif *m* randomly and then implant a random instance *m*′ of *m* to each sequence with a probability *q*. Each instance *m*′ differs from *m* in at most *d* positions; as shown in column 2 of Table 1, the value of *d* under a specific *l* is determined in terms of the challenging problem instance of planted motif search [27]. When generating a random motif instance *m*′ from *m*, the specific Hamming distance *i* (0 ≤ *i* ≤ *d*) between *m*′ and *m* is determined by (8), where *g* is the motif conservation parameter.

Based on this generation method, two groups of simulated data sets are designed, and they can be downloaded at https://sites.google.com/site/feqond/pairmotifchip. For both groups of data sets, *n* is fixed to 200; *q* is fixed to 0.8;* l* is taken as 9, 15, and 21, representing short, medium, and long motif length, respectively. The other settings for the first group of data sets are as follows: *t* is fixed to 600, which is the largest sequence number that MEME-ChIP supports to process; *g* is taken as 0.2, 0.5, and 0.8, representing high, medium, and low conservation, respectively. For the second group of simulated data sets, we vary *t* from 500 to 3000 to obtain different data scales and fix *g* as 0.5.

The used real data are mouse embryonic stem cells (mESC) ChIP-seq data [30]. Each mESC data set, which corresponds to a specific transcription factor, is a set of 200-length sequences with each sequence being a peak region bound by the specific transcription factor. These data sets contain thousands to tens of thousands of sequences, and we used the first 600 sequences to make motif discovery for each data set.

### 3.3. Evaluation

In the experiments, we compare the running time and identification accuracy of different motif discovery algorithms. For the site-level identification accuracy, we evaluate it by using the site-level performance coefficient sPC [29]. When we say a motif site *m*′ is in a set of motif sites *M*, there exists a motif site *m*′′ in *M* such that *m*′ overlaps *m*′′. Let *K* and *P* represent the published motif sites and the predicted motif sites, respectively. Then, site-level true positive (sTP), false negative (sFN), and false positive (sFP) are the number of motif sites in both *K* and *P*, in *K* but not in *P*, and not in *K* but in *P*, respectively. Based on this, sPC is calculated as follows:

(13) $$\begin{array}{c} sPC=\frac{sTP}{sTP+sFN+sFP}. \end{array}$$

For the nucleotide-level identification accuracy, we evaluate it by using the nucleotide-level correlation coefficient (nCC) [31], an integrated assessment of nucleotide-level sensitivity (nSn) and specificity (nSp). The value of nCC ranges from −1 to +1; a high nCC indicates that the predicted motif is more accurate. Let *K* and *P* represent the nucleotide positions covered by the published motif sites and the predicated motif sites, respectively. Then, nucleotide-level true positive (nTP), false negative (nFN), false positive (nFP), and true negative (nTN) are the number of nucleotide positions in both *K* and *P*, in *K* but not in *P*, not in *K* but in *P*, and in neither *K* nor *P*, respectively. Based on this, nCC, nSn, and nSp are calculated as follows:

(14) nCC=nTP×nTN−nFN×nFPnTP+nFNnTN+nFPnTP+nFPnTN+nFNnSn=nTPnTP+nFNnSp=nTNnTN+nFP.

### 3.4. Results on Simulated Data

We select four compared algorithms: MEME-ChIP [23], F-motif [18], PairMotif+ [13], and qPMS9 [16]. MEME-ChIP is a widely used motif discovery algorithm for ChIP-seq data based on PWM. F-motif is a motif discovery algorithm for ChIP-seq data based on word. PairMotif+ is a motif discovery algorithm designed in our previous work. qPMS9 is a recently proposed motif discovery algorithm; it is the best one in exact motif discovery algorithms and can identify motif efficiently in traditional promoter sequences.

All the algorithms are implemented in C or C++. Except for MEME-ChIP, whose results are produced by its web server (http://meme-suite.org/tools/meme-chip), all the algorithms run on the same machine with a 2.67 GHz CPU and a 4 G memory. Each result is the average obtained on three randomly generated data sets with the same settings. Both PairMotifChIP and MEME-ChIP use the default parameters to identify motifs. In executing F-motif, the minimum motif length is set to 8, and the value of *d* is set to 2, 5, and 8 when the length of the identified motif is 9, 15, and 21, respectively. Both PairMotif+ and qPMS9 need specified *l* and *d* of the identified motif. When calculating identification accuracy, the predicated motif sites are needed. For MEME-ChIP, the predicated motif sites are returned by its web server; for other algorithms, the sites are obtained by searching the substring in each input sequence having the smallest Hamming distance from the predicted motif.

For the first group of simulated data sets, the running time, the site-level identification accuracy, and the nucleotide-level identification accuracy are given in Tables 2, 3, and 4, respectively. It can be seen that (i) all these algorithms show good identification accuracy, since large data sets contain sufficient motif information; (ii) PairMotifChIP has the best time performance among these algorithms; namely, it is able to deal with the DNA data set of 600 sequences within one minute; (iii) MEME-ChIP has the second best time performance, and it solves the problem within half an hour; (iv) for other compared algorithms, their running time becomes impractical with the increase of *l* because of exhaustively verifying* l*-length candidate motifs.

For the second group of simulated data sets, whose data scales range from 500 to 3000 sequences, the comparison of different algorithms is shown in Table 5. Here, MEME-ChIP and qPMS9 are not taken as the compared algorithms, as MEME-ChIP allows the processed data set containing at most 600 sequences and qPMS9 has a poor time performance in processing large data sets. It can be seen that (i) the three motif discovery algorithms still have a good identification accuracy; (ii) PairMotifChIP performs slower than F-Motif when *l* is 9, while it is significantly faster than the other two algorithms when *l* is 15 and 21; (iii) the running time of PairMotifChIP increases with the increase of data scale, but it does not depend on the motif length.

Finally, it is necessary to test the method for extracting pairs of *l*-mers because it plays a key role in the time performance of the whole PairMotifChIP algorithm.  Table 6 shows the comparison of the running time of the method in this paper and the method in [26]. When extracting pairs of *l*-mers with Hamming distance no more than *k* in two given *n*-length sequences, although the worst time complexity of the method in this paper is still *O*(*n* ^2^), it has a better time performance in practice. Specifically, the method in this paper is 10 times faster than that in [26], which makes it possible to process more DNA sequences.

### 3.5. Results on Real Data

We test PairMotifChIP's validity of motif discovery on the real ChIP-seq data (i.e., the mESC data). To better show the results, we take our previous algorithm PairMotif+ for comparison. For PairMotifChIP, we use default parameters without any prior information; for PairMotif+, we use the same setting *l* = 13, * d* = 4, and *k* = 2 for each data set.

Table 7 shows the results on these real data. For each data set, we list the name of the specific transcription factor, the published motif, and the running time and predicted motif of the two algorithms. The motifs are shown in the form of sequence logos [32]. PairMotifChIP runs much faster than PairMotif+ on these data. Moreover, PairMotifChIP can successfully find a motif overlapping the published motif for each data set, while PairMotif+ fails to make prediction on the n-Myc, Smad1, STAT3, and Tcfcp2I1 data sets.

## 4. Conclusions

In order to identify motifs in large DNA data sets, we propose a new algorithm, PairMotifChIP, which is a ChIP-tailored version of PairMotif/PairMotif+. The main difference between PairMotifChIP and PairMotif/PairMotif+ is that (i) PairMotifChIP designs a more efficient method for extracting pairs of *l*-mers and (ii) unlike PairMotif/PairMotif+, which obtains motifs by exhaustively verifying candidate motifs generated from extracted pairs of *l*-mers, PairMotifChIP obtains motifs by combining extracted *l*-mers based on clustering methods. The improvements of PairMotifChIP over PairMotif/PairMotif+ are that (i) PairMotifChIP runs much faster when identifying motifs in large data sets and (ii) PairMotifChIP can make motif discovery without any prior information (e.g., the motif length). The executable program of PairMotifChIP is available at https://sites.google.com/site/feqond/pairmotifchip.

It should be noted that, limited by the idea of combining overrepresented substrings, PairMotifChIP may not work well on the traditional promoter sequence data set containing dozens of sequences because of the lack of sufficient motif information.

## Figures and Tables

**Algorithm 1:**
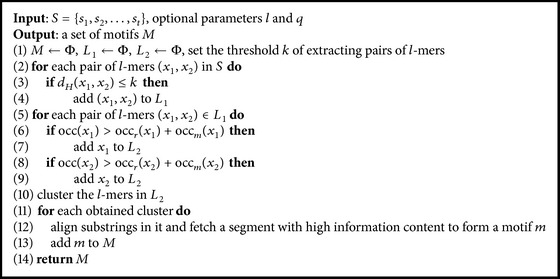
PairMotifChIP.

**Table 1:** Data for probabilistic analysis.

*l*	*d*	*k*	*E*_*k*_	*N*_1_	*N*_2_	occ_*r*_(*x*)	occ_*m*_(*x*)
8	1	0	0.57	284715	12784	2.95	32.00
9	2	0	0.14	69930	511	0.73	1.28
10	2	0	0.04	19980	511	0.19	1.28
11	3	1	0.29	144855	3577	1.54	8.95
12	3	1	0.08	39960	3196	0.42	8.00
13	4	2	0.39	194805	4581	2.08	11.46
14	4	2	0.11	54945	4059	0.60	10.16
15	5	3	0.43	214785	5274	2.30	13.20
16	5	3	0.13	64935	4584	0.70	11.47

**Table 2:** Running time on the first group of simulated data sets.

*l*	*g*	PairMotifChIP	MEME-ChIP	F-motif	PairMotif+	qPMS9
9	0.2	26.3 s	1510.1 s	9.2 s	300.7 s	247.4 s
	0.5	21.3 s	1507.1 s	9.2 s	212.9 s	234.7 s
	0.8	18.7 s	1462.6 s	9.1 s	217.9 s	226.0 s

15	0.2	35.9 s	1325.0 s	16655.1 s	73048.5 s	—
	0.5	25.6 s	1354.9 s	16403.4 s	23549.0 s	—
	0.8	19.5 s	1466.7 s	15982.7 s	845.6 s	—

21	0.2	47.4 s	1425.5 s	—	—	—
	0.5	30.7 s	1148.5 s	—	—	—
	0.8	20.5 s	1349.2 s	—	—	—

*Note*. s: seconds; —: over 24 hours.

**Table 3:** Site-level identification accuracy on the first group of simulated data sets.

*l*	*g*	PairMotifChIP	MEME-ChIP	F-motif	PairMotif+
9	0.2	0.942	0.866	0.942	0.942
	0.5	0.902	0.734	0.902	0.902
	0.8	0.907	*∗*	0.907	0.907

15	0.2	0.995	0.960	0.995	0.995
	0.5	0.969	0.916	0.969	0.969
	0.8	0.936	*∗*	0.936	0.936

21	0.2	1.000	0.947	—	—
	0.5	0.988	0.953	—	—
	0.8	0.981	0.844	—	—

*Note*. —: the result is not obtained because the running time is over 24 hours; ^*∗*^the result is not obtained because motif sites are not provided by MEME-ChIP on the corresponding data sets. The site-level identification accuracy is evaluated by the site-level performance coefficient sPC. Since qPMS9 and F-motif report the same motifs and have the same identification accuracy, the results of qPMS9 are not listed in this table.

**Table 4:** Nucleotide-level identification accuracy on the first group of simulated data sets.

*l*	*g*	PairMotifChIP			MEME-ChIP			F-motif			PairMotif+		
		nCC	nSn	nSp	nCC	nSn	nSp	nCC	nSn	nSp	nCC	nSn	nSp
9	0.2	0.969	0.970	0.999	0.927	0.899	0.999	0.969	0.970	0.999	0.969	0.970	0.999
	0.5	0.947	0.949	0.998	0.849	0.762	0.999	0.947	0.949	0.998	0.947	0.949	0.998
	0.8	0.921	0.929	0.996	*∗*	*∗*	*∗*	0.950	0.951	0.998	0.950	0.951	0.998

15	0.2	0.997	0.997	1.000	0.978	0.997	0.998	0.997	0.997	1.000	0.997	0.997	1.000
	0.5	0.983	0.984	0.999	0.952	0.950	0.997	0.983	0.984	0.999	0.983	0.984	0.999
	0.8	0.965	0.967	0.998	*∗*	*∗*	*∗*	0.965	0.967	0.998	0.965	0.967	0.998

21	0.2	1.000	1.000	1.000	0.969	1.000	0.994	—	—	—	—	—	—
	0.5	0.993	0.994	0.999	0.972	0.955	0.996	—	—	—	—	—	—
	0.8	0.989	0.990	0.999	0.921	0.906	0.996	—	—	—	—	—	—

*Note*. —: the result is not obtained because the running time is over 24 hours; ^*∗*^the result is not obtained because motif sites are not provided by MEME-ChIP on the corresponding data sets. Since qPMS9 and F-motif report the same motifs and have the same identification accuracy, the results of qPMS9 are not listed in this table. Besides the nucleotide-level identification accuracy nCC, the sensitivity nSn and specificity nSp are also listed in this table.

**Table 5:** Running time and identification accuracy on the second group of simulated data sets.

*l*	Sequence #	PairMotifChIP	F-motif	PairMotif+
9	500	14.4 s (0.955)	7.8 s (0.955)	68.1 s (0.628)
	1000	60.6 s (0.945)	17.2 s (0.945)	410.1 s (0.945)
	1500	133.3 s (0.953)	27.8 s (0.953)	989.9 s (0.953)
	2000	231.4 s (0.953)	40.0 s (0.953)	1704.1 s (0.953)
	2500	361.4 s (0.951)	52.8 s (0.951)	3012.7 s (0.951)
	3000	519.2 s (0.955)	67.2 s (0.955)	4307.4 s (0.955)

15	500	17.9 s (0.986)	13581.7 s (0.986)	14394.4 s (0.986)
	1000	74.8 s (0.983)	30293.2 s (0.983)	35172.2 s (0.983)
	1500	150.9 s (0.980)	50102.5 s (0.980)	—
	2000	253.0 s (0.981)	66344.7 s (0.981)	—
	2500	396.9 s (0.982)	—	—
	3000	554.4 s (0.981)	—	—

21	500	22.9 s (0.995)	—	—
	1000	90.5 s (0.996)	—	—
	1500	171.6 s (0.995)	—	—
	2000	277.2 s (0.995)	—	—
	2500	423.8 s (0.996)	—	—
	3000	592.2 s (0.996)	—	—

*Note.* s: seconds; —: over 24 hours. The number after each running time is the corresponding nucleotide-level identification accuracy nCC.

**Table 6:** Running time of methods for extracting pairs of *l*-mers.

Sequence* #*	Method in this paper	Method in [26]
200	2.2 s	23.6 s
400	8.7 s	96.1 s
600	19.7 s	197.9 s
800	34.7 s	331.7 s
1000	54.3 s	518.1 s
1200	78.4 s	741.6 s
1400	109.0 s	1015.4 s
1600	140.5 s	1334.1 s
1800	178.3 s	1731.2 s
2000	223.2 s	2163.5 s

*Note*. s: seconds.

**Table 7:** Results on the mESC data.

Data set	Published motif	PairMotifChIP		PairMotif+	
		Time	Predicted motif	Time	Predicted motif
c-Myc	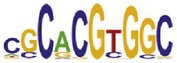	37.2 s	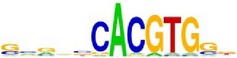	4106.1 s	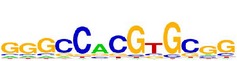
CTCF	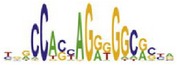	29.1 s	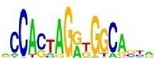	23584.3 s	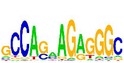
Esrrb	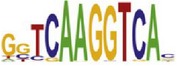	25.6 s	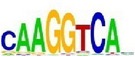	7424.6 s	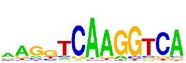
Klf4	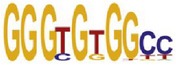	29.3 s	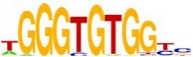	3558.5 s	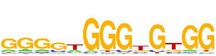
Nanog	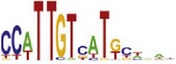	24.3 s	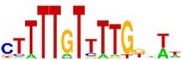	1975.6 s	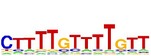
n-Myc	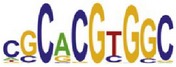	36.3 s	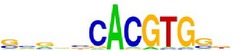	33962.6 s	—
Oct4	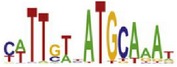	8.9 s	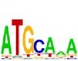	2608.8 s	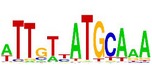
Smad1	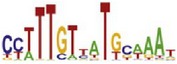	20.3 s	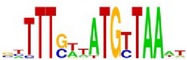	5296.1 s	—
Sox2	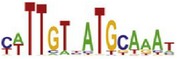	23.1 s	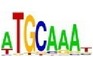	4115.2 s	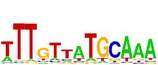
STAT3	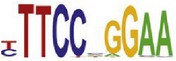	22.9 s	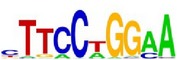	6342.6 s	—
Tcfcp2I1	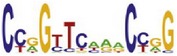	23.5 s	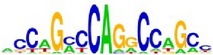	2269.5 s	—
Zfx	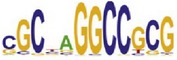	42.2 s	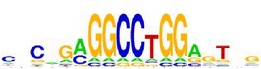	3617.2 s	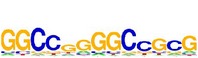

*Note.* —: there is no motif overlapping the published motif in the top ten predicted motifs.

## Notations

*l*: The motif length
*S* = {*s*_1_, *s*_2_, …, *s*_*t*_}: The input DNA data set; each input sequence *s*_*i*_ is an *n*-length string over the alphabet {A, C, G, T}
*t*: The number of input sequences, *t* = |*S*|
*n*: The length of each input sequence
*q*: The probability that each input sequence contains a motif instance
*d*: The maximum number of positions where a motif differs from its instance
*g*: The motif conservation parameter
*l*-mer: An *l*-length string over {A, C, G, T}
*k*: The threshold of extracting pairs of *l*-mers, 0 ≤ *k* < *l*
occ(*x*): The number of *l*-mers in the input sequences with Hamming distance no more than *k* from an *l*-mer *x*
occ_*r*_(*x*): The number of *l*-mers in the input sequences with Hamming distance no more than *k* from an arbitrary *l*-mer *x* in random case; it is calculated by (6)
occ_*m*_(*x*): The number of motif instances in the input sequences with Hamming distance no more than *k* from an arbitrary motif instance *x* in random case; it is calculated by (11)
*s*[*i* ⋯ *j*]: A substring of the string *s* starting from the *i*th position to the *j*th position.
